# Bacteriocin-Nanoconjugates (Bac10307-AgNPs) Biosynthesized from *Lactobacillus acidophilus*-Derived Bacteriocins Exhibit Enhanced and Promising Biological Activities

**DOI:** 10.3390/pharmaceutics15020403

**Published:** 2023-01-25

**Authors:** Arif Jamal Siddiqui, Mitesh Patel, Mohd Adnan, Sadaf Jahan, Juhi Saxena, Mohammed Merae Alshahrani, Abdelmushin Abdelgadir, Fevzi Bardakci, Manojkumar Sachidanandan, Riadh Badraoui, Mejdi Snoussi, Allal Ouhtit

**Affiliations:** 1 Department of Biology, College of Science, University of Ha’il, Ha’il P.O. Box 2440, Saudi Arabia; 2 Department of Biotechnology, Parul Institute of Applied Sciences and Centre of Research for Development, Parul University, Vadodara 391760, India; 3 Department of Medical Laboratory Sciences, College of Applied Medical Sciences, Majmaah University, Al Majmaah 11952, Saudi Arabia; 4 Department of Biotechnology, University Institute of Biotechnology, Chandigarh University, Gharuan, NH-95, Ludhiana-Chandigarh State Hwy, Punjab 140413, India; 5 Department of Clinical Laboratory Sciences, Faculty of Applied Medial Sciences, Najran University, 1988, Najran 61441, Saudi Arabia; 6 Department of Oral Radiology, College of Dentistry, University of Ha’il, Ha’il P.O. Box 2440, Saudi Arabia; 7 Section of Histology-Cytology, Medicine Faculty of Tunis, University of Tunis El Manar, La Rabta-Tunis 1017, Tunisia; 8 Laboratory of Genetics, Biodiversity and Valorization of Bio-Resources (LR11ES41), University of Monastir, Higher Institute of Biotechnology of Monastir, Avenue Tahar Hadda, BP74, Monastir 5000, Tunisia; 9 Department of Biological and Environmental Sciences, College of Arts and Sciences, Qatar University, Doha P.O. Box 2713, Qatar

**Keywords:** bacteria, *L. acidophilus*, silver nanoparticles, cancer, bacteriocins, TEM, protein target

## Abstract

The proteinaceous compounds produced by lactic acid bacteria are called bacteriocins and have a wide variety of bioactive properties. However, bacteriocin’s commercial availability is limited due to short stability periods and low yields. Therefore, the objective of this study was to synthesize bacteriocin-derived silver nanoparticles (Bac10307-AgNPs) extracted from *Lactobacillus acidophilus* (*L. acidophilus*), which may have the potential to increase the bioactivity of bacteriocins and overcome the hurdles. It was found that extracted and purified Bac10307 had a broad range of stability for both temperature (20–100 °C) and pH (3–12). Further, based on Sodium dodecyl-sulfate polyacrylamide gel electrophoresis (SDS–PAGE) analysis, its molecular weight was estimated to be 4.2 kDa. The synthesized Bac10307-AgNPs showed a peak of surface plasmon resonance at 430 nm λmax. Fourier transform infrared (FTIR) confirmed the presence of biological moieties, and transmission electron microscopy (TEM) coupled with Energy dispersive X-Ray (EDX) confirmed that AgNPs were spherical and irregularly shaped, with a size range of 9–20 nm. As a result, the Bac10307-AgNPs displayed very strong antibacterial activity with MIC values as low as 8 μg/mL for *Staphylococcus aureus* (*S. aureus*) and *Pseudomonas aeruginosa* (*P. aeruginosa*), when compared to Bac10307 alone. In addition, Bac10307-AgNPs demonstrated promising in vitro antioxidant activity against 2,2-diphenyl-1-picrylhydrazyl (DPPH) (IC_50_ = 116.04 μg/mL) and in vitro cytotoxicity against human liver cancer cells (HepG2) (IC_50_ = 135.63 μg/mL), more than Bac10307 alone (IC_50_ = 139.82 μg/mL against DPPH and 158.20 μg/mL against HepG2). Furthermore, a protein–protein molecular docking simulation study of bacteriocins with target proteins of different biological functions was also carried out in order to ascertain the interactions between bacteriocins and target proteins.

## 1. Introduction

*Lactobacillus* is a genus of bacteria known as a probiotic and has been recognized as a vital part of human health and well-being since the 18th century. One of the lactobacilli species, *Lactobacillus acidophilus*, has been studied extensively since it was discovered in 1890 and has gained more attention than any of the others [1]. Research has documented that the organism is an intestinal resident of the human body since its inoculation, which is why it is being commercially available as a dietary supplement as well as in many dairy products [2]. As a result of further research carried out over the past century, it has been reported that individuals who consume products containing *L. acidophilus* have a number of significant health benefits. From this perspective, it can be viewed as having the potential to benefit the host by enhancing lactose digestion, treating diarrhea, improving blood lipid chemistry, boosting immunity, and potentially killing cancer cells [3]. In fact, species of *L. acidophilus* are specially equipped with the producing capabilities of bacteriocins and bacteriocins, such as the compounds lactacin B, lactacin F, acidocin A, and acidocin B, which are at the core of many industrial and medicinal applications [4,5].

The bacteriocins are a miscellaneous group of proteinaceous compounds produced by different types of bacteria that usually exhibit antibacterial activity against pathogenic bacteria belonging to different genera [6]. There are various uses of bacteriocins in the healthcare sector including as biopreservatives for food and as an antibacterial agent for use in biomedical applications, which have attracted the attention of researchers [7,8]. However, to date, nisin and pediocin PA-1 are the only two antimicrobial peptides approved by the Food and Drug Administration (FDA) for use as biopreservatives [9]. Even though various researchers all over the world have characterized and studied a large number of bacteriocins, there are a number of limitations associated with their application, including degradation by proteases, narrow antimicrobial spectrum, restricted production, high dosage needed at the same time, etc. [10].

In the last few years, the combination of nanotechnology with biotechnology has become one of the most important areas of research and development in the medical and pharmaceutical industries due to its potential impact. This technology has also emerged as a promising solution for overcoming some of the limitations of bacteriocins that have been mentioned above, as well as providing a ray of hope for the future. It is well known that nanoparticles synthesized through biological processes are environment-friendly and safe to be used. There have been a number of studies conducted in recent years on the application of metallic nanoparticles, specifically silver nanoparticles (AgNPs), which are highly conductive, magnetic, thermal, and possess different biological properties [11,12]. The bioactive potential of bacteriocin and silver nanoparticles have been reported, which includes antimicrobial, antioxidant, and anticancer potentials. Therefore, in order to increase the bioactivity of bacteriocins, nanotechnology can be useful for conjugating bacteriocins with metal nanoparticles, which may have the potential to increase the bioactivity of bacteriocins [9]. In the present study, bacteriocin Bac10307 was extracted and purified from *L. acidophilus*, and its physicochemical properties were characterized. The synthesis of Bac10307-AgNPs, their characterization via different biophysical methods, and evaluation of their in vitro bioactive potential (antibacterial, antioxidant and cytotoxicity against cancer cells) was carried out. Moreover, the bioactive potential of the reported bacteriocins of *L. acidophilus* were further assessed via in silico molecular docking analysis.

## 2. Materials and Methods

### 2.1. Bacterial Strains and Growth Conditions

A standard bacterial strain of *L. acidophilus* MTCC-10307 was obtained from the Microbial Type Culture Collection (IMTECH, Chandigarh, India). Bacteria were grown and maintained on MRS (De Man, Rogosa and Sharpe) agar plates (HiMedia^®^, Mumbai, India). The pure strain was kept at 4 °C for future use.

### 2.2. Extraction and Purification of Bacteriocin from L. acidophilus

The active culture of *L. acidophilus* was inoculated into MRS broth and incubated at 30 °C for 24–48 h. After incubation, a supernatant was collected using the centrifugation of the grown culture at 10,000 rpm for 15 min at 4 °C. In order to neutralize the effect of organic acid in the collected supernatant, the pH of the supernatant was adjusted to 6.0 by using 1N NaOH solution, and later the filtration of the neutralized supernatant was performed using 0.22 μm membrane filter. Then, a partial purification step involved infusing the collected supernatant with ammonium sulfate salt in the 80% saturation range and continuous stirring on ice, followed by incubating at 4 °C overnight to precipitate proteins. In the next steps, the pellets were collected via centrifugation at 10,000 rpm at 4 °C for 15 min. Then, the obtained pellet was resuspended in a phosphate buffer solution (0.02 M) with pH 7.0. The collected pellet represented crude bacteriocins. The crude bacteriocins were dialyzed overnight at 4 °C against the same buffer to remove salt and other impurities. Lastly, the antibacterial properties of the collected bacteriocins were assessed against different strains of bacteria [13].

### 2.3. Analysis of Antibacterial Activity of Partially Purified Bacteriocin-Bac10307

#### 2.3.1. Bacterial Strains and Growth Conditions

The antibacterial activity of partially purified bacteriocin Bac10307 was carried out against different bacterial pathogens such as, *Staphylococcus aureus* (MTCC96), *Pseudomonas aeruginosa* (MTCC741), *Bacillus subtilis* (MTCC121) and *Escherichia coli* (MTCC 9537). A sterile MHB (HiMedia^®^, Mumbai, India) was used to grow and maintain all strains of bacteria, which were incubated at 30 °C for 24 h in a shaking condition (120 rpm).

#### 2.3.2. Agar Well Diffusion Assay

The agar well diffusion assay was carried out as an initial test. After growing the bacterial strains overnight in MHB, adjustment in the turbidity of culture was carried out using sterile normal saline solution matching the 0.5 McFarland standard (10^8^ CFU/mL). In the following step, 100 μL of each bacterial culture was distributed evenly on the plates, and using a sterile cork borer, wells were made at the center of the plates. Each well was inoculated with 50 μL of the partially purified bacteriocin Bac10307 solution, chloramphenicol (50 μg/mL), and sterile distilled water. Incubation of the plates was carried out at 37 °C for 24 h. As a sign of antibacterial activity, a zone of inhibition was observed [14].

### 2.4. Assessment of Stability of Bacteriocin-Bac10307

The Bac10307 were examined for stability against a variety of physicochemical factors [15].

#### 2.4.1. Impact of Temperatures

The effect of temperatures ranging from 20 to 100 °C on bacteriocin Bac10307 activity was evaluated by incubating bacteriocin Bac10307 at the respective temperatures for 20 min [16]. An antibacterial activity via agar well diffusion assay was performed in order to analyze the effects of temperature on bacteriocin Bac10307 bioactivity.

#### 2.4.2. Impact of pH

The effect of different pH (2–10) on bacteriocin Bac10307 activity was evaluated by incubating bacteriocin Bac10307 at respective pH [17]. In order to adjust the pH of the bacteriocin culture solution, 1N HCl and 1N NaOH solution was used. An antibacterial activity via well diffusion assay was performed in order to analyze the effects of temperature on bacteriocin Bac10307 bioactivity.

#### 2.4.3. Impact of Enzymes

An examination of the effects of various proteolytic enzymes, such as proteinase-K, trypsin, and α-amylase, on bacteriocin Bac10307 activity was evaluated by incubating bacteriocin Bac10307 with the respective enzymes [18]. Each enzyme (1 mg/mL) in separate batches was mixed with the extracted bacteriocin Bac10307 and incubated at 37 °C for 2 h. For the purpose of denaturing the enzyme, further heating was performed on the mixture was performed at 95 °C for 10 min at the end of the incubation procedure. Using the well diffusion assay, the antibacterial activity of bacteriocin was assessed.

### 2.5. Molecular Mass Determination of Bacteriocin Bac10307 Using SDS–PAGE

The sodium dodecyl sulfate–polyacrylamide gel electrophoresis (SDS–PAGE) was used for the analysis of extracted bacteriocin Bac10307 to determine the molecular mass [19]. Coomassie Brilliant Blue R-250 stain was used to stain the gels after electrophoresis. A protein ladder (245–3.5 kDa) was used to determine the molecular weights of bacteriocin-Bac10307.

### 2.6. Synthesis of Bacteriocin-10307-AgNPs

Using a method described by Sidhu and Nehra (2021) [20], bacteriocin-derived silver nanoparticles were synthesized. Filter-sterilized solutions of AgNO_3_ (2 mM) were mixed with partially purified bacteriocin-10307 (5:45 mL) at room temperature for 1 h under UV light. Afterwards, the mixture was centrifuged for 30 min at 12,000 rpm to stop the reaction and remove unbound metal ions and bacteriocin-10307. After centrifugation, the supernatant discarded carefully, and the pellet was twice rinsed in ethanol and resuspended in sterile de-ionized water. As a result of this process, AgNPs were obtained with Bac10307, and as a consequence, they were referred to as Bac10307-AgNPs.

### 2.7. Characterization of Bac10307-AgNPs

The characterization of Bac10307-AgNPs was carried out via UV–vis spectrophotometry, transmission electron microscopy (TEM), energy-dispersive X-ray spectroscopy (EDX), and Fourier-transform infrared (FT-IR) spectroscopy.

#### 2.7.1. UV–Vis Spectrophotometer

The formation of Bac10307-AgNPs was initially characterized by spectrophotometric analysis. In order to determine the UV–visible spectra of the synthesized Bac10307-AgNPs, the spectra were scanned in a range of wavelengths between 400 and 700 nm with a resolution of 1 nm via a UV–vis spectrophotometer (Shimadzu, Japan) [21].

#### 2.7.2. Transmission Electron Microscopy (TEM) and Energy-Dispersive X-Ray (EDX)

The size and shape of the synthesized Bac10307-AgNPs were characterized by using TEM (Tecnai 20, Philips, Holland). For analysis, Bac10307-AgNPs were equally stained with phosphotungistic acid solution (0.5%) and then fixed on copper grids, dried, and then imaged with a CCD camera in a TEM [22]. Then, EDX analysis (INCA, Oxford Instruments, UK) of the Bac10307-AgNPs was carried out at 20 KeV to accurately define the elemental constitution of the formed particles.

#### 2.7.3. Fourier-Transform Infrared (FT-IR) Analysis

The infrared absorbance of Bac10307-capped AgNPs were measured by using a FTIR spectrophotometer (Bruker^®^, Billerica, MA, USA) in the spectral range of 400 to 4000 cm^−1^. The FTIR analysis enabled us to deduce that there are functional groups that play a role in stabilizing and reducing synthesized nanoparticles [23].

### 2.8. Screening of Antibacterial Activity of Bac10307-AgNPs

#### 2.8.1. Agar Well Diffusion Assay

In order to evaluate the antibacterial activity of Bac10307-AgNPs and AgNPs synthesized via the Brust method against different bacterial pathogens, the agar well diffusion assay was carried out using the method describe above [14].

#### 2.8.2. Assessment of Minimum Inhibitory Concentration (MIC)

In order to determine the MIC value of Bac10307-AgNPs against test bacterial strains, a broth dilution method was employed [24]. An inoculum was prepared by taking an active culture of each bacterium and growing it in MHB for overnight. In 96-well plates (100 μL per well), synthesized Bac10307-AgNPs were diluted two-fold, ranging from 256.0 to 2.0 μg/mL. Following this, 20 μL of each bacterial culture (10^8^ CFU/mL) was added and incubated for 24 h at 37 °C. In the next step, MICs were determined by measuring the concentrations at which observable growth was inhibited. The positive control contained only bacteria inoculated without any synthesized Bac10307-AgNPs in the culture, while the negative control contained only the media.

### 2.9. Screening of In Vitro Antioxidant Activity of Bac10307-AgNPs

In order to measure the capability of Bac10307 and Bac10307-AgNPs to scavenge free radicals, the antioxidant activity was measured against DPPH as a measure of their scavenging activity. Then, 10 μL of different concentrations of Bac10307 and Bac10307-AgNPs (2–256 μg/mL) was added to 200 μL of the 0.1 mM DPPH solution in a 96-well plate. The blank consists of 200 μL of DMSO and 10 μL of a compound at various concentrations. The plates were incubated in the dark for a period of 30 min. A microplate reader (iMark, BioRad, USA) was used to measure the decolorization at 495 nm at the end of the incubation. A reaction mixture containing 20 μL of deionized water was used as a control mixture. With respect to the control, the scavenging activity was expressed as a % inhibition [25].

DPPH Scavenging activity = ((Abs Control − Abs Sample)/AbsControl) × 100

### 2.10. Screening of In Vitro Cytotoxicity Assay (MTT Assay) of Bac10307-AgNPs

In order to determine the cytotoxic activity of Bac10307 and Bac10307-AgNPs, they were tested on Hep-G2 cells derived from human liver cancer. Dulbecco’s Modified Eagle’s Medium (DMEM) (MP Biomedicals, Eschwege, Germany) was used to grow Hep-G2 cells in flasks (25 cm^2^) at 37 °C in humidified atmospheres with 5% CO_2_ supplemented with 10% fetal bovine serum and 10,000 units/mL penicillin and 5 mg/mL streptomycin antibiotic solution (Hi-Media, Mumbai, India). When cells achieved 80% confluency, they were seeded at a density of 10^4^ cells per well in 96-well plates and incubated under the same conditions. Following the staining of the cells with Trypan Blue (Hi-Media^®^, Mumbai, India) (0.4%), the viability of the cells was investigated using a hemocytometer. After that, the cells were exposed to various concentrations of Bac10307 and Bac10307-AgNPs (2–256 μg/mL) for 48 h. Afterwards, the plate was removed from the incubator, and the media containing Bac10307 and Bac10307-AgNPs were aspirated. Thereafter, 200 μL of medium containing 10% MTT reagent (MP Biomedicals, Eschwege, Germany) was added to each well, and the plates were again incubated at 37 °C for an additional 3 h under a humidified atmosphere (5% CO_2_). To dissolve the formazan crystals, 100 μL of DMSO (Merck, Darmstadt, Germany) was added in the following step after the medium was removed. In order to measure the amount of formazan crystal in the sample, an ELISA reader (EL10A, Biobase, China) was used to measure the absorbance at the 570 nm and 630 nm wavelengths. Using the dose–response curve for the respective cell line, the percentage of growth inhibition (IC_50_) was determined by subtracting the background and blank. The positive control used for this assay was the cisplatin [26].

### 2.11. Molecular Docking (MD) Assays

Crystal structures of different target proteins, such as DNA gyrase B (PDB: 6F86.pdb) for antibacterial activity, NADPH oxidase for antioxidant activity (PDB: 2CDU.pdb), and VEGFR2 (PDB: 2OH4.pdb) for anticancer activity, were fetched from the Protein Data Bank (RCSBPDB). Following the retrieval of protein crystal structures, reported bacteriocins of *L. acidophilus*, such as acidocin A, acidocin B, and lactacin F, were predicted from the AlphaFold protein structure database. The ClusPro protein–protein docking server (https://cluspro.bu.edu, accessed on 17 October 2022) was used for the simulation of molecular docking [27,28,29]. To confirm the binding position between bacteriocins and the target proteins, the docking results were visualized in the PyMOL version 2.5.2 and Discovery Studio version 21.1.0.20298.

### 2.12. Statistical Analysis

All the results are expressed as mean ± SD of the number of experiments performed. A significance test was carried out among the treatments by two-way ANOVA followed by Bonferroni post tests at *p* < 0.05. Statistical analysis was conducted with software GraphPad Prism 8.0.

## 3. Results

### 3.1. Bacteriocin-Bac10307: Extraction, Purification, and Antibacterial Activity Analysis

**Figure 1:**
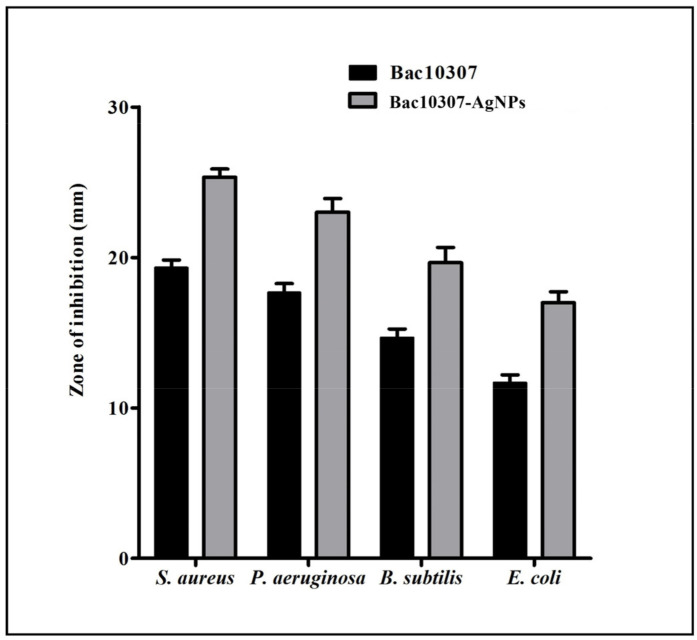
Antibacterial activity (zone of inhibition) of Bac10307 and Bac10307-capped AgNPs against *S. aureus*, *P. aeruginosa*, *B. subtilis,* and *E. coli*. The test was carried out in triplicate, and the data represent the mean ± SD, n = 3.

The isolation and purification of bacteriocin Bac10307 was carried out from the *L. acidophilus* bacteria. Among the tested pathogenic test strains that were used as a part of this study, bacteriocin Bac10307 exhibited antibacterial properties against all the tested strains viz. *S. aureus*, *P. aeruginosa*, *B. subtilis,* and *E. coli*, which indicates that Bac10307 demonstrates antibacterial activity against Gram-positive as well as Gram-negative bacteria. There was a maximum zone of inhibition observed for *S. aureus,* followed by *B. subtilis*, *P. aeruginosa,* and *E. coli* (Figure 1).

### 3.2. Characterization of Purified Bacteriocin-Bac10307

**Figure 2:**
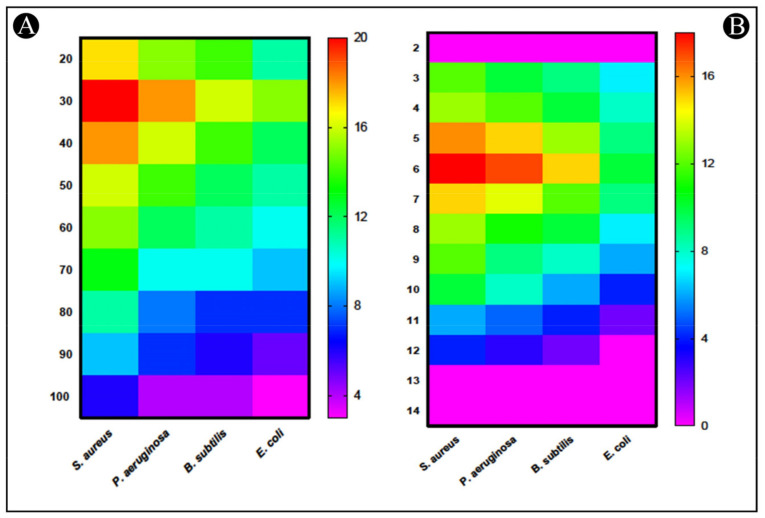
Effect of (**A**) temperature, and (**B**) pH on the activity of Bac10307 extracted from *L. acidophilus*.

In a study to evaluate the effect of temperature on the antibacterial activity of Bac10307, results showed that Bac10307 was stable at temperatures ranging from 20 to 100 °C. A high level of antibacterial response was observed at temperatures between 30 °C and 40 °C, followed by a slight decline in antibacterial activity as temperatures rose from 30 to 100 °C (Figure 2A). Although the antimicrobial potential of the culture had decreased, it still exhibited high temperature stability despite the decrease in the zone of inhibition.

From the results of a study conducted to assess how pH affects the antibacterial activity of Bac10307, it showed antibacterial activity between pH ranges of 3 to 12 except against *E. coli*. It was observed that Bac10307 maintained the highest antibacterial activity at pH 6, thus showing the highest stability at this pH as well (Figure 2B).

From the results of the study conducted to assess whether the antibacterial effect was entirely a result of the purified Bac10307, rather than any other molecular moiety present, it has been found that, after treating Bac10307 with proteinase-K and trypsin, antibacterial activity was completely lost, while activity remained unchanged in the presence of α-amylase.

**Figure 3:**
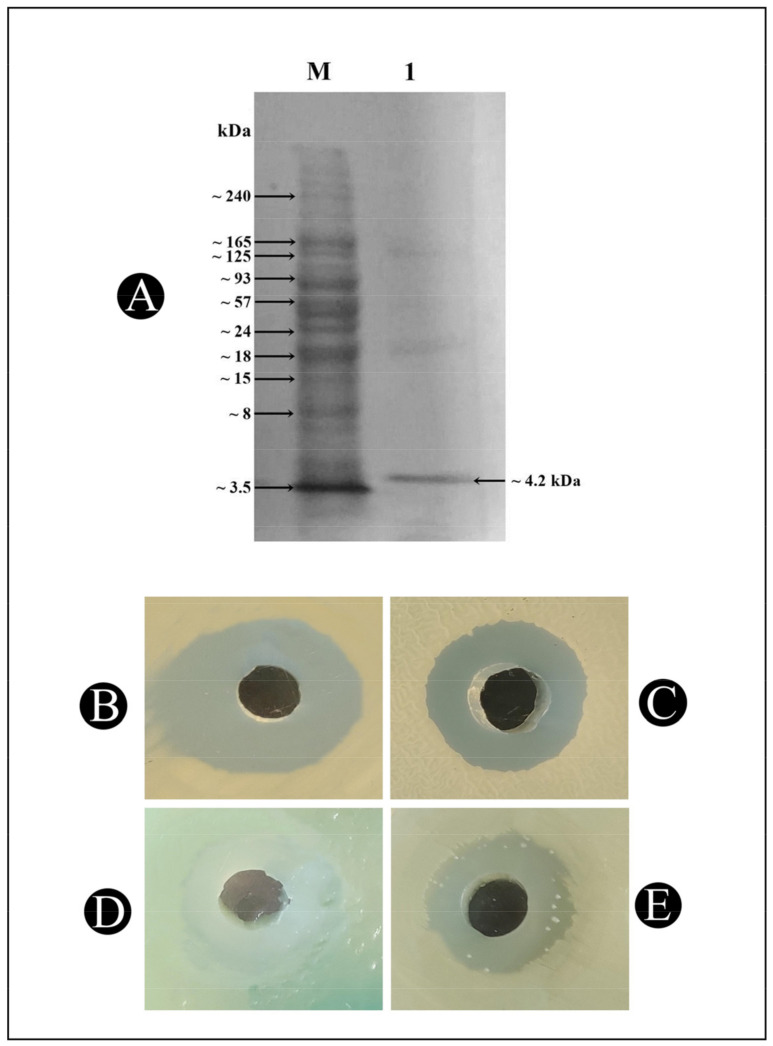
(**A**) Determination of molecular weight of bacteriocin by SD-SPAGE. Lane M contains the molecular-weight marker. Lane 1 contains bacteriocin from *L. acidophilus*. (**B**) Zone of inhibition of antibacterial activity of Bac10307-AgNPs against *S. aureus*, (**C**) zone of inhibition of antibacterial activity of Bac10307-AgNPs against *P. aeruginosa*, (**D**) zone of inhibition of antibacterial activity of Bac10307-AgNPs against *B. subtilis*, (**E**) zone of inhibition of antibacterial activity of Bac10307-AgNPs against *E. coli*.

According to the SDS–PAGE gel electrophoresis analysis, the purified bacteriocin Bac10307 has a molecular weight around 4.2 kDa, along with few faint bands in the lane (Figure 3A).

### 3.3. Synthesis and Characterization of Bac10307-AgNPs

**Figure 4:**
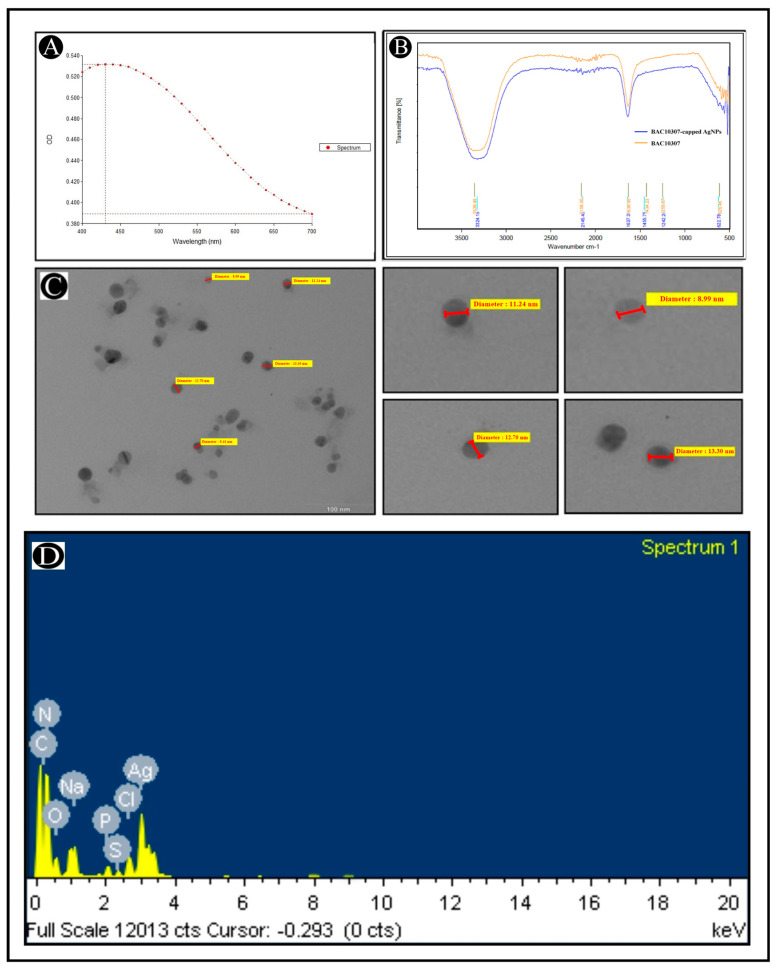
Characterization of Bac10307-AgNPs. (**A**). UV-visible absorption spectra of synthesized Bac10307-AgNPs. (**B**). FT-IR pattern of Bac10307 and Bac10307-AgNPs. (**C**). Morphological analysis of synthesized Bac10307-AgNPs with variable diameter using TEM analysis. (**D**). EDX analysis.

Following the characterization of Bac10307, the synthesis of Bac10307-AgNPs was carried out. As a result of the addition of Bac10307 to the AgNO_3_ solution, a color change was observed from light yellow to reddish brown, which provided an indication that Bac10307-AgNPs were synthesized. Different analytical techniques were used for the purpose of determining the size, morphology, and stability of the synthesized Bac10307-AgNPs. The UV–visible spectroscopy analysis revealed the absorption spectrum had a peak maximum at 430 nm, indicating that synthesized Bac10307-AgNPs were successfully synthesized (Figure 4A).

It is known that AgNPs exhibit an absorption peak between 400 and 500 nm in the UV–visible spectrum. Generally, AgNPs appear to have an absorption band due to the free electrons in them, which are caused by their mutual vibration of light wave incidences, resulting in a Surface Plasmon Resonance (SPR) absorption band. The synthesized AgNPs were also subjected to spectroscopic measurements two weeks after synthesis, and the spectroscopic data displayed no significant variation in the spectroscopic results, which was an indication that the synthesized AgNPs were stable over time.

For the identification of functional groups involved during the synthesis of AgNPs, a FTIR study was conducted. In light of the findings of this study, it appears that the structure of Bac10307 did not undergo any major changes as a result of its affiliation with AgNPs during the synthesis process. In addition, an almost similar spectrum was observed for Bac10307-AgNPs as for Bac10307 (Figure 4B). The peaks for Bac10307-AgNPs were observed at 3339.49, 2159.00, 1636.95, 1434.23, and 1255.67 cm^−1^. These peaks indicate the presence of OH and NH_2_ groups, C≡C stretch of alkynes, C–O and C–N stretching and peptide linkage in amides, and C–H stretching.

In the TEM analysis, Bac10307-AgNPs were shown to be spherical nanoparticles with irregular shapes of various sizes and their dispersion was good without any obvious agglomeration. In terms of size, the AgNPs synthesized were between 9 and 20 nm (Figure 4C). In addition, an EDX analysis was carried out to determine the overall composition of elements in the reaction mixture of extracted bacteriocin and silver nitrate after the reactions had been completed. The results revealed a strong signal in the silver region at 3 KeV and confirms the formation of silver nanoparticles along with other elements (Figure 4D).

### 3.4. Antibacterial Activity of Bac10307 and Synthesized Bac10307-AgNPs

As compared to Bac10307 alone, Bac10307-AgNPs displayed enhancement in the activity. Antibacterial activity of the synthesized Bac10307-AgNPs was 1.35-, 1.31-, 1.34-, and 1.54-fold greater than Bac10307 (Figure 1). As a next step, MIC and MBC analysis was further carried out in the presence of increasing dilutions of Bac10307-AgNPs. A visual analysis as well as measurements of the growth pattern at 600 nm was performed. The values of MIC were 8 μg/mL for *S. aureus* and *P. aeruginosa*, 16 μg/mL for *B. subtilis,* and 64 μg/mL for *E. coli,* and MBC values were observed to be two times higher than the MIC values (Table 1).

### 3.5. In Vitro Antioxidant Activity of Synthesized Bac10307-AgNPs

**Figure 5:**
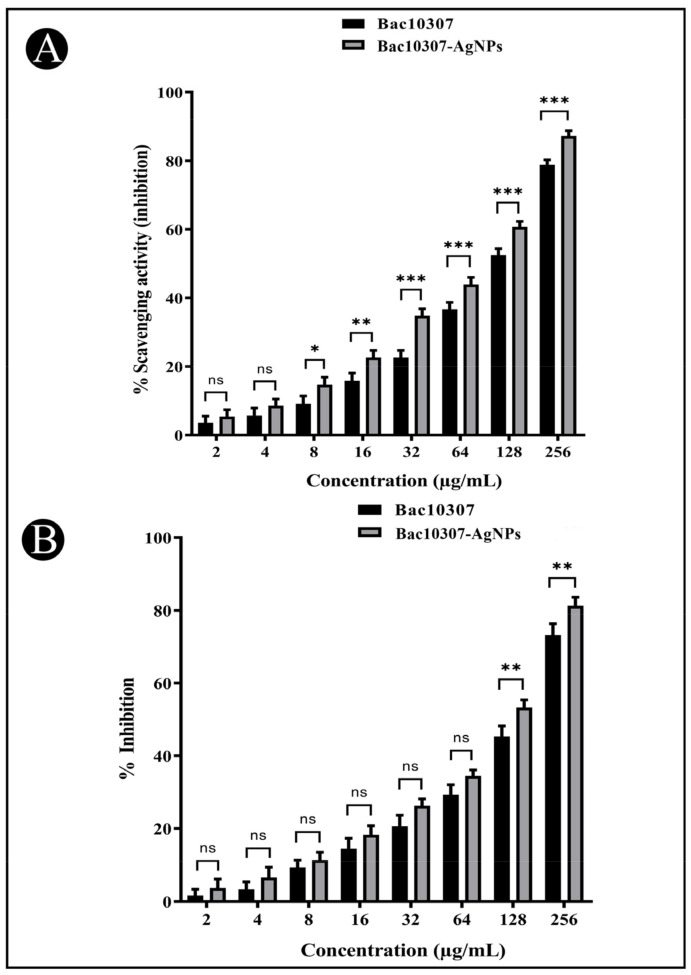
(**A**). Antioxidant activity of Bac10307 and Bac10307-AgNPs against DPPH free radicals. (**B**) Cytotoxicity of Bac10307 and Bac10307-AgNPs against HepG2 cancer cells. The test was carried out in triplicate, and the data represent the mean ± SD, n = 3. Significance; ns > 0.05, * *p* < 0.05, ** *p* < 0.01, *** *p* < 0.001.

A study of the antioxidant potential of synthesized Bac10307-AgNPs has been conducted to assess their inhibitory effect on DPPH free radicals. As a result, the synthesized Bac10307-AgNPs were observed to exhibit dose-dependent free radical scavenging activity against DPPH (IC_50_ = 116.04 μg/mL) viz. increase in concentration (2–256 μg/mL) enhanced the antioxidant potentiality more than Bac10307 alone did (IC_50_ = 139.82 μg/mL) (Figure 5A).

### 3.6. In Vitro Cytotoxicity of Synthesized Bac10307-AgNPs

**Figure 6:**
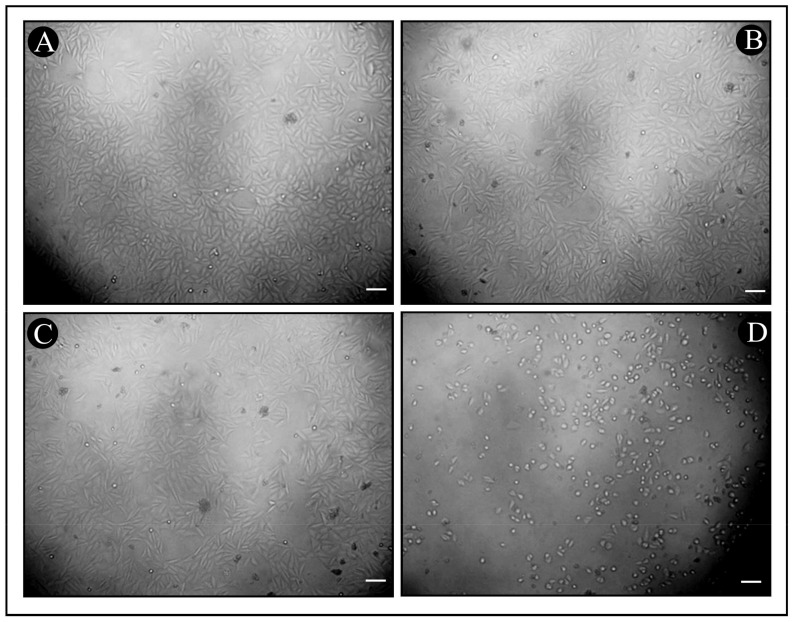
Morphological analysis of HepG2 cells under inverted microscope after treatment with different concentrations of Bac10307-AgNPs with morphological changes. (**A**) Untreated, (**B**) 4 μg/mL, (**C**) 64 μg/mL, (**D**) 256 μg/mL (magnification 20×, scale bar 50 μm).

Potential cytotoxic properties of synthesized Bac10307-AgNPs was evaluated via MTT assay against human liver cancer cells (Hep-G2). The results indicated that Bac10307-AgNPs inhibited the proliferation of Hep-G2 cancer cells (IC_50_ = 135.63 μg/mL) more than Bac10307 alone did (IC_50_ = 158.20 μg/mL) in a dose-dependent manner (Figure 5B and Figure 6A–D).

### 3.7. Molecular Docking Analysis

**Figure 7:**
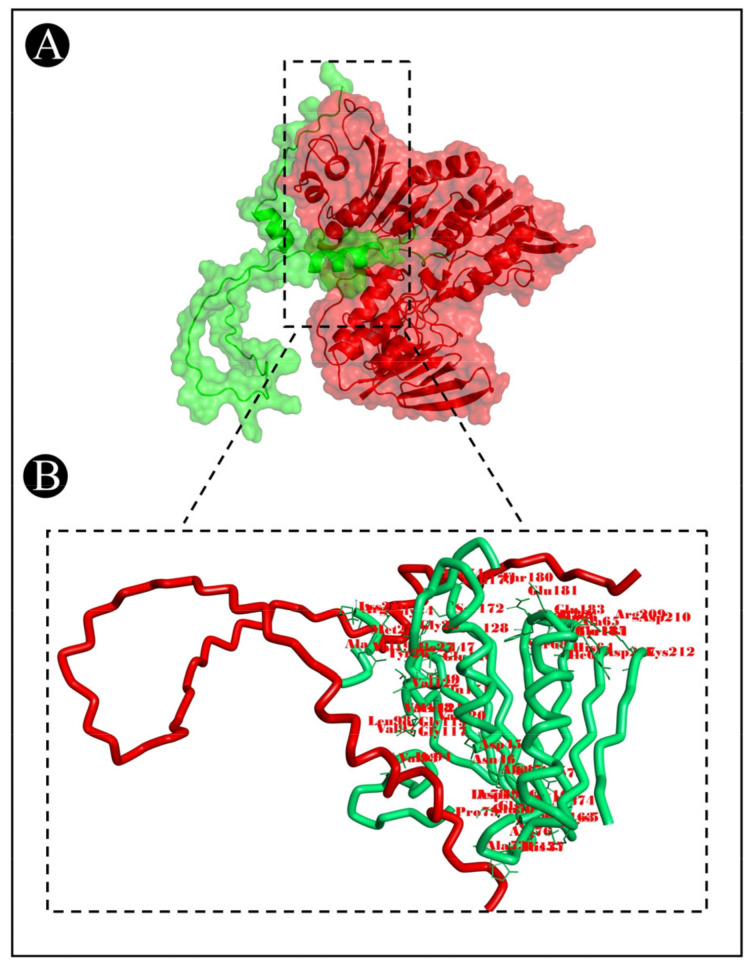
Docking representation of the acidocin A and 2CDU complex. (**A**) The binding interface of the complex, (**B**) the binding interaction between the amino acids.

**Figure 8:**
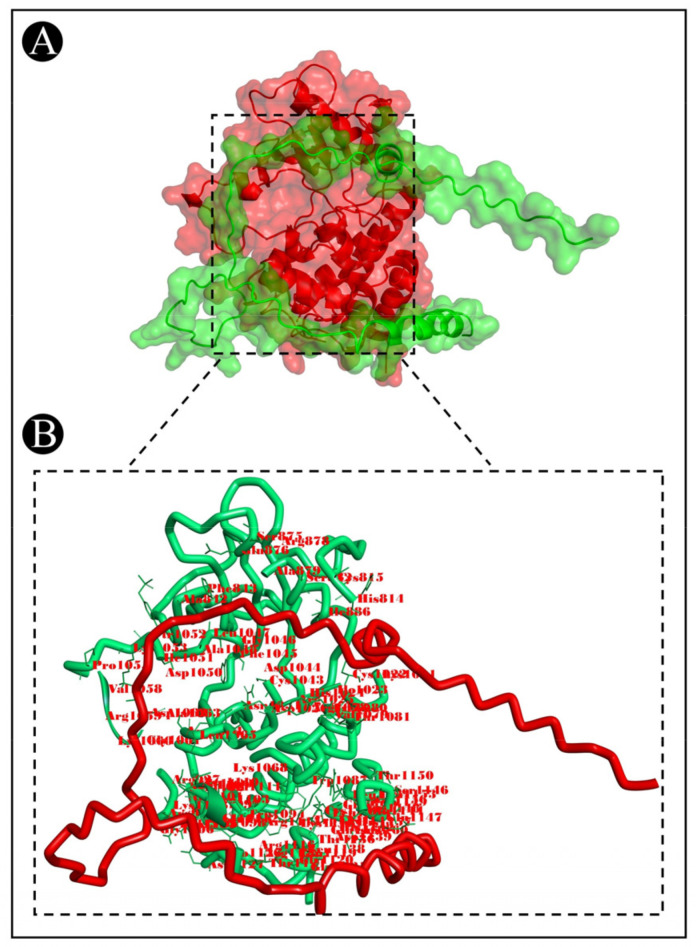
Docking representation of acidocin A and 2OH4 complex. (**A**) The binding interface of the complex, (**B**) the binding interaction between the amino acids.

**Figure 9:**
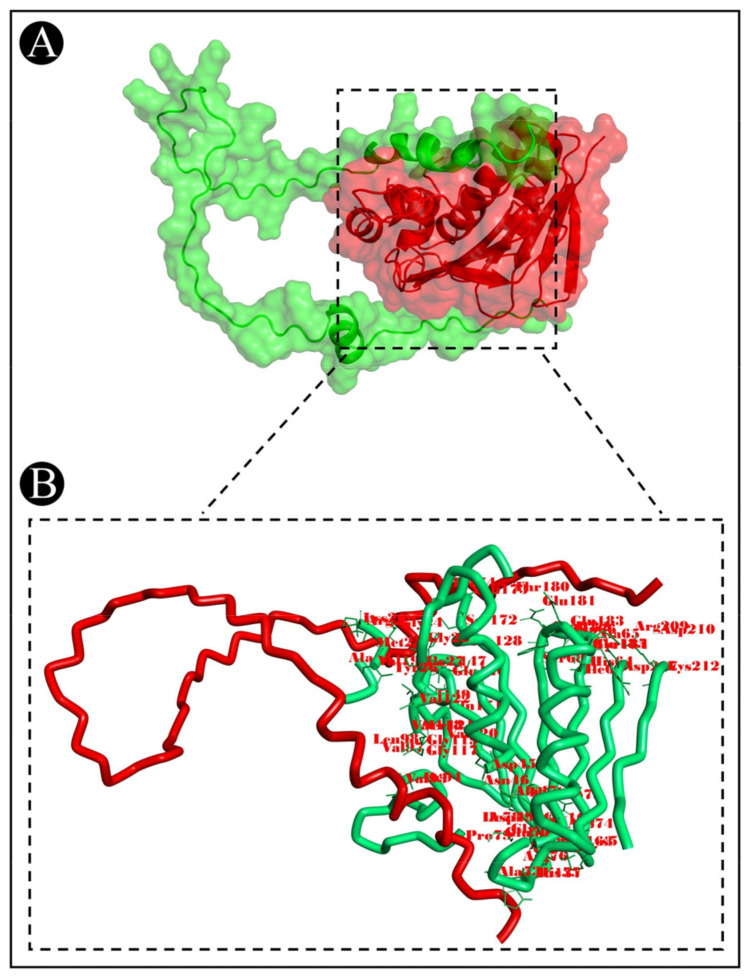
Docking representation of the acidocin A and 6F86 complex. (**A**) The binding interface of the complex, (**B**). the binding interaction between the amino acids.

To predict the protein–protein binding modes and affinities, ClusPro 2.0 was used to pair the bacteriocins of *L. acidophilus* with the selected target proteins. The ClusPro scores are based on searching for the lowest free binding energy for native site. A summary of the results is represented in Table 2. It shows the size of each cluster (number of members), the cluster center’s energy score (i.e., its most neighbouring structure), and its lowest energy structure (Supplementary Table S1). ClusPro has been also used to generate molecular docking complexes that can be used to analyze the interactions between the amino acid residues of target proteins and bacteriocins (Figure 7, Figure 8 and Figure 9).

## 4. Discussion

The antibacterial activity of bacteriocins makes them a very promising alternative as an additive to the currently used antibiotics combatting the epidemic of different bacterial infections that are sweeping across the globe today. Apart from antimicrobial activity, some of them have also been reported for their multifunctional properties, such as antioxidant and anticancer activity [30,31,32,33,34]. Thus, it is speculated that some of them could constitute a potential source of new biologically active agents in the future. Despite these promising advantages, nisin continues to be the only bacteriocin that is considered safe by the Food and Drug Administration (FDA) and is now used as a food preservative internationally [35]. There are several factors that contribute to the limited availability of bacteriocins in the market as preservatives and antimicrobials, including their high production costs [36], loss of their activity due to proteolytic enzymes [36], their adverse interconnection with other food constituents [37], change in the physical and chemical properties at the time of different food-processing stages [38], insufficient recovery by traditional purification methods of these compounds, and the limited scale of activity detected for most of the tested bacteriocins towards pathogenic bacteria [39]. It has been demonstrated in recent years that optimizing the production conditions, the purification method, the combination with other antimicrobial agents, and the hurdle technology approach can all contribute to solving some of these problems related to bacteriocins [40]. A potential approach to maximizing the effectiveness of bacteriocins is through the use of nanotechnology, which has been shown to be an effective means to overcome the limitations of these peptides [41]. Thus, the purpose of the present study was to elucidate the current applications of nanotechnology in improving the properties and bioactive potential of bacteriocins in order to improve its efficacy.

In the present study, bacteriocin Bac10307 was extracted from *L. acidophilus* and partially purified before being used. A partially purified bacteriocin Bac10307 was observed to possess antibacterial properties against *S. aureus*, *B. subtilis*, *P. aeruginosa,* and *E. coli*, indicating that it exhibits antibacterial properties against both Gram-positive and Gram-negative bacteria. It has also been reported that similar results have been found in a few previous studies. In a study carried out by De Giani et al. (2019) [42], plantsaricin P1053 produced by the *L. plantarum* PBS067 strain was found to show broad spectrum antimicrobial activity against both Gram-positive and Gram-negative bacteria. Similarly, in the study conducted by Danilova et al. (2019) [43], antimicrobial peptides isolated from *L. plantarum* were shown to inhibit the growth of *S. aureus*, *E. coli,* and *P. aeruginosa* in vitro, confirming previous findings.

Among the factors that influence the activity of bacteriocins, temperature is an important one. As shown in the present study, bacteriocin extracted from *L. acidophilus* was quite active even after being exposed to high temperatures, indicating that it is a heat-stable protein. In other studies, Abo-Amer (2007) [44] and Fatima and Mebrouk (2013) [45] have made similar observations that highlight the usefulness of *L. plantarum* bacteriocin as a preservative procedure for food due to its high-temperature tolerance. Similarly, bacteriocin Lac-B23 has been reported to have its antibacterial activity even after being heated for 30 min at 121 °C in order to sustain its antibacterial response [18]. In addition, another study reported that at 50, 70, and 80 °C, the levels of bacteriocins produced by *L. bulgaricus*, *L. acidophilus*, and *L. helveticus* remained constant. In spite of this, the bacteriocins from *L. acidophilus* and *L. bulgaricus* were the only ones still effective at 100 °C [46]. As a result, it can be concluded that each bacteriocin behaves differently depending on the conditions it is exposed to. It should be noted that the bacteriocins obtained in our study were capable of maintaining their activity even at very high temperatures (80–100 °C), thus supporting their potential application in the food industry.

A second factor that affects the antibacterial activity of bacteriocins is pH. During the present study, Bac10307 was found to exhibit antimicrobial activity in the pH range of 3–12 with the highest antimicrobial activity being detected at pH 6. A similar trend has also been reported when optimizing pH values for L23, a novel bacteriocin produced by *L. plantarum*-J23. A pH stability range of 2.0 to 12.0 was observed for the bacteriocin. According to another study, plantsaricin LPL-1, which is produced by *L. plantarum*, was found to be stable in a pH range between 2 and 10, and its antibacterial activity decreased with an increase in pH value until a total loss of activity was observed at pH 11 [15]. The bacteriocin L23 has also been shown to retain 90% of its activity up to pH 7.0 in a previous study [18].

Apart from the effect of temperature and pH, it has been determined that the antibacterial properties of Bac10307 are exclusively due to the protein itself, not by any other molecular moiety following treatment with proteinase-K, trypsin, and α-amylase. Bac10307 did not display any sensitivity towards α-amylase, which indicated that the antibacterial activity of it did not change in the presence of this enzyme. Therefore, we can infer that the presence of hydrogen peroxide and glycoproteins does not have any effect on the antibacterial response. Even though this fact had been noted, the antibacterial activity of the Bac10307 could not recover after treatment with trypsin and proteinase-K, possibly due to the enzymes and their role in destroying the active site of Bac10307 during the process. Based on its sensitivity to both proteolytic enzymes, it was concluded that Bac10307 is a proteinaceous in nature. Previously, Zhang et al., (2018) [18] reported that the bacteriocin Lac-B23 was not sensitive to papain or pepsin, but it lost antimicrobial activity after its treatment with proteinase-K, trypsin, and proteinase-E. Similarly, the antimicrobial activity of the bacteriocin obtained by *L. murinus* AU06 was also completely lost following treatment with chymotrypsin, proteinase K, trypsin, and pepsin [16].

SDS–PAGE gel electrophoresis of the purified Bac10307 was performed, and the molecular mass was determined to be around 4.2 kDa; [47] reported the isolation of sheep raw milk cheese bacteriocin-producing *L. plantarum* strains used in a similar study as ours, and using SDS–PAGE gel electrophoresis and MALDI–TOF analysis, the bacteriocin produced from these strains was determined to have a size of 4.8 kDa. A similar report was published by Song (2009) [48], in which they reported the synthesis of plantaricin ZJ5, which had a molecular mass of 2.5 kDa. It has also been discovered that there are a variety of novel plantaricins discovered from *L. plantarum* in recent years, including the plantaricin ASM1 (5 kDa) [49], plantaricin C19 (3.8 kDa) [50], plantaricin 163 (3.5 kDa) [51], plantaricin Y (4.2 kDa) [52], plantaricin LPL-1 [53], and Lac B-23 [18].

A number of factors have been identified that limit the activity of natural bacteriocins, including the rapid degradation of the bacteriocins within a few days in the environment as well as a high effective concentration accompanied by a low yield [54]. As one of the most successful methods of overcoming most of these problems, nanotechnology can be considered one of the most modern methods. According to Lazzari et al., (2012) [55], the stability of nanoparticles in biological fluids is excellent, in addition to their efficient antimicrobial properties due to the high surface area of the particles. Because of these advantages, it seems like a good idea to incorporate nanotechnology into bacteriocin encapsulation to enhance its properties [56]. As a consequence, using the nanotechnology approach, we produced bacteriocin-derived silver nanoparticles using the bacteriocin extracted from the culture and compared the bioactive potential of the bacteriocin with the nanoparticle formulations. It appears from the results of our study that Bac10307-AgNPs were synthesized, which was confirmed by UV–vis spectroscopy. There is a strong band of absorption at 430 nm that was observed in the synthesized Bac10307-AgNPs as a result of their SPR properties. There is a strong possibility that this was caused by the stimulation of longitudinal plasmon vibrations and the reduction of AgNO*_3_* ions as well [57]. A FT-IR study was conducted in order to determine the functional properties of nanoparticles by identifying the associated functional groups. An analysis of the FT-IR spectrum revealed a number of absorption peaks, including those associated with the OH and NH*_2_* groups, stretching of alkynes, C–O and C–N stretching and peptide linkage in amides, and C–H stretching. In the analysis of images obtained by the TEM of Bac10307-AgNPs, it can be seen that the majority of the particles were crystalline in nature and irregularly shaped or spherical in shape, with varying sizes. It has been reported in previous studies that the TEM analysis results of the spherical Bac23-capped AgNPs were irregularly shaped and of different sizes [20]. EDX is an analytical technique used to determine the elemental composition of a sample and to characterize its chemical composition, which relies on the interaction of some source of X-ray excitation with the sample. According to the EDAX spectral analysis of Bac10307-AgNPs preparations, there was a strong silver signal that is typical for metallic silver. However, weaker signals related to other atoms were also detected, which can be attributed to the bacteriocin bound to the AgNPs. Based on the results of the antibacterial response, Bac10307-AgNPs showed an enhancement in antibacterial performance compared to Bac10307 and AgNPs alone in terms of antibacterial effectiveness. Taking these results into account, it can be suggested that there may be a synergistic or enhanced effect of bacteriocin and AgNPs. Considering the recent findings, it might be possible to use AgNPs as an adjuvant for the treatment of various infectious diseases caused by Gram-negative and Gram-positive bacteria. Hence, the findings of our study support the claim that AgNPs are capable of exerting significant antibacterial activity, which can be used to enhance the effectiveness of existing antibiotics against Gram-negative and Gram-positive bacteria. Earlier research conducted by few researchers has also indicated that bacteriocin-derived AgNPs increase the antibacterial activity [58,59,60]. According to Thirumurugan et al., 2013 [60], an increase in activity was observed upon the combination of gold nanoparticles with bacteriocins. Anti-listerial activity also increased when gold nanoparticles were combined with pediocin–LAP conjugates, as reported by Singh et al., 2018 [61]. In addition, Morales-Avila et al., 2017 [62], found that ubiquicidin-conjugated SNPs showed improved antimicrobial activity compared to silver or gold nanoparticles alone in their study. Various free radicals are linked to oxygen, including reactive oxygen species (ROS) and reactive nitrogen species (RNS), which are able to react with molecules other than oxygen. By generating these free radicals, oxidative stress is induced, causing proteins, lipids, and nucleic acids to be damaged. Thus, free radicals are believed to play a role in a wide range of diseases, such as aging, inflammatory diseases (such as arthritis, a variety of respiratory diseases, and vasculitis in adults), neurological disorders (such as Alzheimer’s disease, Parkinson’s disease), ischemic diseases (stroke, heart attacks, and intestinal ischemia), and various types of cancer [25]. There are certain substances called antioxidants that act as defenses against free radicals. A DPPH assay was used to evaluate the in vitro antioxidant activity of Bac10307-AgNPs, and the results revealed that the Bac10307-AgNPs could scavenge radicals to a larger extent compared with Bac10307 alone.

At the present time, in spite of all the advances in oncology, cancer remains one of the most life-threatening diseases around the world [63]. In the world, liver cancer is ranked as the third-leading cause of death from cancer worldwide. In 2020, an estimated 830,180 people died around the world as a result of this disease [64]. Currently, radiation therapy, chemotherapy, and surgery are the three most common methods for treating cancer. This treatment has a range of side effects that are known to have a negative impact on human health. Therefore, there is a need for the findings of an alternative treatments or compounds which can treat cancer [65]. In this regard, the in-vitro cytotoxic potential of synthesized Bac10307-AgNPs was also investigated in this study for their anticancer potential against liver cancer cells. Based on the cytotoxic activity results of Bac10307-AgNPs, it has been found that they significantly inhibit the viability of liver cancer cells more than Bac10307 alone.

Moreover, the use of computer-aided drug discovery approaches is becoming more prevalent and is becoming one of the most useful tools for detecting medications derived from natural resources. For pharmaceutical and technology research, computational prediction models are essential for guiding the process of selecting the most appropriate methodology. These models have also been applied to the in silico prediction of drug behavior, including pharmacokinetics, pharmacology, and toxicology [66]. As a strategy for developing and testing pharmaceuticals, molecular docking has proven to be an efficient and cost-effective technique at present. Using this approach, it is possible to generate data on the interactions between drugs and receptors. These data can then be used for predicting the binding orientation of drugs to their targets. It has also been demonstrated that, using this method, a molecule can placed non-covalently into the binding site of an object macromolecule which facilitates systematic investigation, resulting in the binding of every ligand to the specific active sites of the object macromolecule [67,68].

The protein–protein docking technique is one of the most popular molecular modelling techniques because it is based on the use of computer algorithms and techniques in order to predict a complex’s orientation and position as molecules are arranged together [69]. Using the ClusPro web server, simulations of molecular docking were conducted to determine the possibility of bacteriocin–protein interactions in the present study. The docking events resulted in the formation of stable complexes and the determination of lowest energy. Docking models are ranked by ClusPro in relation to the size of the conformation cluster to which they belong. As part of the docking energy calculation, ClusPro provides two types of docking energy: the core energy of a cluster of conformations and the lowest energy in the cluster as a whole [70]. There are a number of biomolecular applications in which protein–protein docking can be utilized, such as exploration of the conformational properties of enzymes, interactome predictions, recognition of molecules, dimerization of proteins, synthesis of specific probes for target proteins, aggregation of amyloid plaques, prediction of protein interactions, design of disease-fighting peptides, and designing vaccines [71].

Bacterial DNA gyrase is a type IIA DNA topoisomerase that plays a very important role in the replication and transcription processes of bacterial DNA [72]. The NADPH oxidase (nicotinamide adenine dinucleotide phosphate) enzymes are multi-subunit protein complexes. It is a membrane-bound protein whose primary function is to transfer electrons across the plasma membrane to molecular oxygen, producing the superoxide anion and reactive oxygen species (ROS), such as hydrogen peroxide and hydroxyl radicals. Most ROS are produced by the activity of NADPH oxidase, which is the most prevalent ROS production process [73,74,75,76]. In addition, in order to promote cancer angiogenesis as well as metastasis, VEGFR2 (vascular endothelial growth factor receptor 2) tyrosine kinase receptor plays an important role. Upon activation by VEGF, which is the ligand of VEGFR2, VEGFR2 appears to promote the formation of new blood vessels in its neighboring tissues, thereby facilitating the delivery of growth factors, nutrients, and oxygen for the proliferation, migration, metastasis, and survival of cancer cells. In many types of cancer, including liver cancer, VEGF- and VEGFR2-mediated metastasis contributes to the aggressive nature of the cancer and leads to high mortality rates [77,78]. Therefore, all three proteins are being considered as highly promising targets for respective biological activity, and the molecular docking study was carried out to examine the binding interactions of known bacteriocins of *L. acidophilus* with them, which confirm our finding of the bacteriocins that possess different biological activity. Furthermore, the results of in silico analysis findings also point to the possible mechanism of action of bacteriocin molecules in terms of targeting specific proteins, and this could be investigated further in order to design bacteriocins that target specific biological activities.

## 5. Conclusions

In this study, bacteriocin Bac10307 was extracted from the lactic acid bacteria *L. acidophilus*, and a single-step synthesis of Bac10307-AgNPs was performed in order to increase the bioactivity of bacteriocins. After being exposed to a range of temperatures and pH conditions, different levels of bioactivity were retained, and the molecular weight was determined to be 4.2 kDa. As compared to Bac10307 alone, Bac10307-AgNPs displayed better antibacterial activity against Gram-positive and Gram-negative bacteria, in vitro antioxidant activity against DPPH free radicals, and in vitro cytotoxic activity against liver cancer cells (HepG2) due to their small size and greater stability. Consequently, this nano-preparation could be applied efficiently in many practical applications, such as in the food industry or in the medical industry, as long as it is carefully evaluated for its safety before being used. However, the antioxidant and cytotoxic effects of Bac10307 and Bac10307-AgNPs have been observed in vitro in the present study, for which further research is needed to see whether the Bac10307 and Bac10307-AgNP are capable of manifesting similar effects in vivo.

## Figures and Tables

**Table 1:** Antibacterial activity of synthesized Bac10307 and Bac10307-AgNPs.

Test Organisms	Zone of Inhibition (mm)			MIC (μg/mL) Bac10307-AgNPs	MBC (μg/mL) Bac10307-AgNPs
	Bac10307	AgNPs	Bac10307- AgNPs		
*S. aureus*	19.66	11.66	25.33	8	16
*P. aeruginosa*	17.33	10.33	23.00	8	16
*B. subtilis*	14.66	8.33	19.66	16	32
*E. coli*	11.66	7.66	17.00	64	128

**Table 2:** Interacting active site residues of acidocin A with different target proteins.

Proteins	Receptor-Ligand	Interection Type	Distance
**2CDU-Acidocin A**	A:LYS78:HZ2–A:ASP282:OD2	Salt Bridge;Attractive Charge	1.76041
	A:LYS78:HZ3–A:ASP282:OD1	Salt Bridge;Attractive Charge	1.75112
	A:CYS133:HN–A:HIS81:NE2	Conventional Hydrogen Bond	2.43145
	A:ILE160:HN–A:PHE80:O	Conventional Hydrogen Bond	2.31372
	A:GLY244:HN–A:HIS81:O	Conventional Hydrogen Bond	1.88916
	A:ALA300:HN–A:PHE76:O	Conventional Hydrogen Bond	2.81082
	A:THR301:HN–A:GLY74:O	Conventional Hydrogen Bond	2.55908
	A:ARG305:HH11–A:TRP71:O	Conventional Hydrogen Bond	1.81072
	A:ARG305:HH21–A:TRP71:O	Conventional Hydrogen Bond	1.84157
	A:ARG431:HH12–A:SER3:O	Conventional Hydrogen Bond	2.70789
	A:PHE433:HN–A:ILE5:O	Conventional Hydrogen Bond	2.04607
	A:SER3:HN–A:GLU366:OE1	Conventional Hydrogen Bond	2.29836
	A:SER7:HG–A:PRO432:O	Conventional Hydrogen Bond	1.84543
	A:GLN9:HE22–A:ASP422:OD1	Conventional Hydrogen Bond	2.12445
	A:THR79:HN–A:PRO298:O	Conventional Hydrogen Bond	2.73643
	A:THR79:HG1–A:PRO298:O	Conventional Hydrogen Bond	1.85808
	A:SER41:CB–A:GLY77:O	Conventional Hydrogen Bond	3.70135
	A:ARG431:CD–A:SER3:O	Carbon Hydrogen Bond	3.15632
	A:HIS81:CE1–A:LEU241:O	Carbon Hydrogen Bond	3.13598
	A:LYS187:NZ–A:PHE80	Pi-Cation	3.51826
	A:ARG305:NH1–A:TRP71	Pi-Cation	4.77402
	A:SER7:CA–A:PHE433	Pi-Sigma	3.67277
	A:ILE243:C,O;GLY244:N–A:PHE80	Amide-Pi Stacked	3.93853
	A:ALA300–A:LYS78	Alkyl	4.9093
	A:ILE438–A:ILE5	Alkyl	4.68217
	A:LYS10–A:MET420	Alkyl	4.61562
	A:PHE367–A:ILE2	Pi-Alkyl	5.07639
	A:TRP71–A:VAL304	Pi-Alkyl	5.08256
	A:TRP71–A:ARG305	Pi-Alkyl	4.70863
	A:TRP71–A:ARG305	Pi-Alkyl	4.06273
	A:TRP71–A:ARG308	Pi-Alkyl	5.17719
	A:PHE76–A:LEU330	Pi-Alkyl	4.80412
	A:HIS81–A:PRO117	Pi-Alkyl	4.38714
	A:HIS81–A:LEU132	Pi-Alkyl	5.41817
**6f86-Acidocin A**	A:MET1:N–A:ASP210:OD2	Attractive Charge	5.4936
	A:ARG22:HH11–A:LEU59:O	Conventional Hydrogen Bond	1.77015
	A:ARG22:HH21–A:SER58:OG	Conventional Hydrogen Bond	1.72382
	A:TYR26:HH–A:ASP64:OD2	Conventional Hydrogen Bond	1.97086
	A:ASN46:HD22–A:ALA72:O	Conventional Hydrogen Bond	1.98784
	A:ASN46:HD22–A:THR73:O	Conventional Hydrogen Bond	2.63274
	A:ARG76:HH12–A:GLY77:O	Conventional Hydrogen Bond	1.8161
	A:ARG136:HH11–A:PHE76:O	Conventional Hydrogen Bond	2.53417
	A:ARG136:HH11–A:LYS78:O	Conventional Hydrogen Bond	2.17681
	A:ARG136:HH21–A:LYS78:O	Conventional Hydrogen Bond	1.72262
	A:THR180:HG1–A:SER7:OG	Conventional Hydrogen Bond	1.87972
	A:SER3:HN–A:ARG209:O	Conventional Hydrogen Bond	2.36379
	A:SER3:HG–A:ARG209:O	Conventional Hydrogen Bond	1.86756
	A:GLN9:HE21–A:GLU174:OE2	Conventional Hydrogen Bond	2.01259
	A:LYS10:HN–A:GLN128:OE1	Conventional Hydrogen Bond	2.17849
	A:ALA18:HN–A:ASP14:O	Conventional Hydrogen Bond	1.93332
	A:ALA18:HN–A:LYS15:O	Conventional Hydrogen Bond	2.65481
	A:SER21:HN–A:ALA18:O	Conventional Hydrogen Bond	2.35624
	A:GLY23:HN–A:ALA18:O	Conventional Hydrogen Bond	1.89654
	A:LYS24:HZ3–A:TYR26:OH	Conventional Hydrogen Bond	1.63416
	A:TYR26:HN–A:LYS24:O	Conventional Hydrogen Bond	2.39531
	A:LYS62:HZ1–A:TYR26:O	Conventional Hydrogen Bond	2.61388
	A:LYS62:HZ2–A:TYR26:O	Conventional Hydrogen Bond	2.68718
	A:LYS62:HZ3–A:MET25:O	Conventional Hydrogen Bond	1.66846
	A:LEU68:HN–A:LEU98:O	Conventional Hydrogen Bond	2.64427
	A:THR73:HG1–A:ASP49:OD1	Conventional Hydrogen Bond	1.98276
	A:THR79:HG1–A:ARG76:O	Conventional Hydrogen Bond	1.81635
	A:ARG76:CD–A:GLY77:O	Carbon Hydrogen Bond	3.35609
	A:MET4:CA–A:GLU181:OE2	Carbon Hydrogen Bond	3.26235
	A:TRP71:CD1–A:VAL97:O	Carbon Hydrogen Bond	3.75115
	A:GLU50:OE1–A:PHE76	Pi-Anion	3.4452
	A:ALA18–A:ILE61	Alkyl	4.4781
	A:VAL118–A:LEU65	Alkyl	4.41069
	A:LYS62–A:MET25	Alkyl	5.37497
	A:ALA72–A:VAL120	Alkyl	5.07682
	A:ALA75–A:PRO79	Alkyl	5.06784
	A:ALA75–A:ILE94	Alkyl	4.19361
	A:TYR26–A:LYS62	Pi-Alkyl	4.48685
	A:TYR26–A:LYS24	Pi-Alkyl	4.99044
	A:TRP71–A:VAL97	Pi-Alkyl	4.62974
	A:TRP71–A:VAL97	Pi-Alkyl	5.0319
	A:PHE76–A:ILE78	Pi-Alkyl	5.21439
	A:PHE80–A:ARG76	Pi-Alkyl	4.69087
**2OH4-Acidocin A**	A:ARG1078:HH11–A:ASP14:OD1	Salt Bridge;Attractive Charge	1.77815
	A:ARG1122:HH22–A:ASP64:OD2	Salt Bridge;Attractive Charge	1.82447
	A:LYS24:HZ1–A:ASP1044:OD1	Salt Bridge;Attractive Charge	2.0004
	A:LYS24:HZ3–A:ASP1044:OD1	Salt Bridge;Attractive Charge	2.03938
	A:ARG55:HH21–A:GLU1112:OE2	Salt Bridge;Attractive Charge	1.89818
	A:LYS56:HZ1–A:GLU1111:OE1	Salt Bridge;Attractive Charge	1.69962
	A:LYS56:HZ3–A:GLU1111:OE2	Salt Bridge;Attractive Charge	1.80997
	A:LYS78:HZ3–A:GLU1156:OE2	Salt Bridge;Attractive Charge	1.71364
	A:ARG1078:NH2–A:ASP14:OD2	Attractive Charge	2.75179
	A:ARG1122:NH1–A:ASP64:OD1	Attractive Charge	4.71658
	A:ARG55:NH1–A:ASP1110:OD2	Attractive Charge	5.55384
	A:ARG55:NH1–A:GLU1112:OE1	Attractive Charge	2.72487
	A:ARG878:HH11–A:TYR27:OH	Conventional Hydrogen Bond	1.773
	A:ARG927:HH11–A:SER38:O	Conventional Hydrogen Bond	3.0835
	A:ARG927:HH21–A:SER38:O	Conventional Hydrogen Bond	1.74776
	A:ILE1023:HN–A:SER21:O	Conventional Hydrogen Bond	1.98064
	A:ARG1025:HH11–A:SER21:O	Conventional Hydrogen Bond	1.79986
	A:ARG1025:HH21–A:SER21:OG	Conventional Hydrogen Bond	1.96334
	A:ARG1025:HH21–A:GLY22:O	Conventional Hydrogen Bond	2.07187
	A:LYS1060:HZ2–A:LYS36:O	Conventional Hydrogen Bond	1.63582
	A:GLY1061:HN–A:THR35:O	Conventional Hydrogen Bond	2.45869
	A:ALA1063:HN–A:THR35:OG1	Conventional Hydrogen Bond	2.01064
	A:LYS1108:HZ1–A:ILE49:O	Conventional Hydrogen Bond	2.21003
	A:LYS1108:HZ3–A:GLY51:O	Conventional Hydrogen Bond	1.71447
	A:ARG1115:HH12–A:LYS56:O	Conventional Hydrogen Bond	1.81567
	A:ARG1116:HH11–A:GLN57:OE1	Conventional Hydrogen Bond	1.89055
	A:ARG1116:HH21–A:GLN57:OE1	Conventional Hydrogen Bond	1.9005
	A:ARG1116:HH22–A:SER58:O	Conventional Hydrogen Bond	2.01369
	A:ARG1122:HE–A:ASP64:OD1	Conventional Hydrogen Bond	2.45807
	A:GLY1143:HN–A:ASP64:OD2	Conventional Hydrogen Bond	2.04712
	A:LYS24:HZ1–A:HIS1024:NE2	Conventional Hydrogen Bond	2.30731
	A:LYS24:HZ2–A:HIS1024:O	Conventional Hydrogen Bond	1.64274
	A:HIS33:HN–A:ASP1050:O	Conventional Hydrogen Bond	2.89181
	A:THR35:HG1–A:ARG1059:O	Conventional Hydrogen Bond	2.54504
	A:LYS36:HZ1–A:VAL1107:O	Conventional Hydrogen Bond	1.69429
	A:ARG55:HN–A:GLU1112:OE1	Conventional Hydrogen Bond	2.65589
	A:LYS56:HN–A:GLU1112:OE1	Conventional Hydrogen Bond	2.47201
	A:GLN57:HN–A:GLU1112:OE1	Conventional Hydrogen Bond	1.99761
	A:SER58:HG–A:GLU1119:OE2	Conventional Hydrogen Bond	1.94121
	A:LEU59:HN–A:GLU1119:OE1	Conventional Hydrogen Bond	2.04219
	A:ILE61:HN–A:GLU1119:O	Conventional Hydrogen Bond	1.97082
	A:GLN63:HE21–A:GLY1120:O	Conventional Hydrogen Bond	2.03591
	A:LEU68:HN–A:ASP1139:OD2	Conventional Hydrogen Bond	2.48216
	A:LYS78:HZ1–A:GLN1163:OE1	Conventional Hydrogen Bond	1.98774
	A:LYS78:HZ2–A:ASN1160:OD1	Conventional Hydrogen Bond	1.69053
	A:SER58:CA–A:GLU1119:OE1	Carbon Hydrogen Bond	2.9455
	A:LYS24:NZ–A:HIS1024	Pi-Cation	3.6145
	A:ASP64:OD2–A:HIS1142	Pi-Anion	4.79084
	A:VAL1058:CG1–A:HIS33	Pi-Sigma	3.82332
	A:LYS1021–A:ILE20	Alkyl	4.85838
	A:ALA1101–A:LEU39	Alkyl	4.05593
	A:PRO1149–A:LEU68	Alkyl	5.33682
	A:HIS1142–A:LEU65	Pi-Alkyl	5.30386
	A:TYR27–A:ALA879	Pi-Alkyl	4.63121
	A:TRP40–A:PRO1105	Pi-Alkyl	4.91592
	A:TRP71–A:PRO1149	Pi-Alkyl	4.42062

## Data Availability

Not applicable.

## Supplementary Materials

The following supporting information can be downloaded at: https://www.mdpi.com/article/10.3390/pharmaceutics15020403/s1, Table S1: Interacting active site residues of other bacteriocins with different target proteins.
