# Supplementary material for: Bacteriocin-Nanoconjugates (Bac10307-AgNPs) Biosynthesized from Lactobacillus acidophilus-Derived Bacteriocins Exhibit Enhanced and Promising Biological Activities
Source: Pharmaceutics. 2023 Jan 25;15(2):403. doi: 10.3390/pharmaceutics15020403 (PMC9967518; doi:10.3390/pharmaceutics15020403)
Supplement: Supplementary file 1 [file pharmaceutics-15-00403-s001.zip › pharmaceutics-2048247-supplementary.pdf]

Table S1: Interacting active site residues of other bacteriocins with different target proteins.

Protein	**Receptor-Ligand**	**Interection Type**	**Distance**
**2CDU-Acidocin B**	A:ALA300:HN - A:GLN7:OE1	Conventional Hydrogen Bond	2.6848
	A:THR301:HN - A:GLN7:OE1	Conventional Hydrogen Bond	1.95499
	A:ARG305:HE - A:ALA57:O	Conventional Hydrogen Bond	3.04807
	A:ARG305:HH21 - A:TYR2:O	Conventional Hydrogen Bond	2.03629
	A:ARG305:HH22 - A:ALA57:O	Conventional Hydrogen Bond	1.84058
	A:TRP3:HE1 - A:SER327:O	Conventional Hydrogen Bond	1.94698
	A:TRP45:HE1 - A:TYR435:OH	Conventional Hydrogen Bond	1.97034
	A:PRO43:CD - A:ASP397:O	Carbon Hydrogen Bond	3.0818
	A:ALA44:CB - A:PHE429	Pi-Sigma	3.67554
	A:ALA48 - A:LEU424	Alkyl	4.73006
	A:ALA49 - A:VAL404	Alkyl	4.13372
	A:ALA52 - A:VAL421	Alkyl	4.72078
	A:ALA55 - A:VAL404	Alkyl	4.72187
	A:HIS396 - A:PRO43	Pi-Alkyl	5.4283
	A:TRP45 - A:VAL404	Pi-Alkyl	4.80352
	A:TRP45 - A:MET423	Pi-Alkyl	4.53761
	A:TRP45 - A:LEU436	Pi-Alkyl	5.17648
**2CDU-Lactacin F**	A:ARG305:HH11 - A:ALA33:O	Conventional Hydrogen Bond	2.01119
	A:ARG305:HH21 - A:ALA33:O	Conventional Hydrogen Bond	1.83044
	A:ARG308:HH11 - A:ASN25:O	Conventional Hydrogen Bond	1.7866
	A:ARG308:HH11 - A:VAL26:O	Conventional Hydrogen Bond	2.19157
	A:ARG308:HH21 - A:ASN25:O	Conventional Hydrogen Bond	1.91878
	A:LYS331:HN - A:CYS44:O	Conventional Hydrogen Bond	1.97064
	A:THR38:HG1 - A:GLN400:OE1	Conventional Hydrogen Bond	1.92953
	A:THR42:HG1 - A:SER399:OG	Conventional Hydrogen Bond	1.92953
	A:ALA47:HN - A:GLU163:OE2	Conventional Hydrogen Bond	2.09852
	A:TYR55:HH - A:LEU60:O	Conventional Hydrogen Bond	2.14601
	A:LYS75:HZ1 - A:LEU71:O	Conventional Hydrogen Bond	1.72707
	A:PRO46:CD - A:GLU163:OE2	Carbon Hydrogen Bond	3.30143
	A:HIS54:CE1 - A:HIS10:O	Carbon Hydrogen Bond	3.05448
	A:LYS318:NZ - A:TRP22	Pi-Cation	4.82835
	A:VAL304:CG1 - A:HIS54	Pi-Sigma	3.70403
	A:HIS54 - A:PHE14	Pi-Pi Stacked	5.07033
	A:HIS54 - A:TYR62	Pi-Pi Stacked	4.07917

	A:TYR62 - A:TYR55	Pi-Pi T-shaped	5.93686
	A:PRO24 - A:LYS75	Alkyl	5.42808
	A:ALA300 - A:VAL50	Alkyl	3.6542
	A:ALA300 - A:ALA53	Alkyl	4.47499
	A:VAL319 - A:VAL26	Alkyl	4.64205
	A:VAL26 - A:LEU312	Alkyl	5.01538
	A:ALA33 - A:VAL304	Alkyl	4.81276
	A:CYS44 - A:LEU46	Alkyl	3.93851
	A:PRO46 - A:LEU330	Alkyl	4.84717
	A:ALA47 - A:LEU46	Alkyl	4.53793
	A:CYS48 - A:LEU46	Alkyl	5.26647
	A:VAL50 - A:LEU40	Alkyl	4.47585
	A:ALA51 - A:LEU40	Alkyl	4.53413
	A:ALA53 - A:VAL304	Alkyl	4.28383
	A:ARG74 - A:PRO24	Alkyl	5.31453
	A:HIS10 - A:VAL50	Pi-Alkyl	5.04111
	A:PHE14 - A:PRO57	Pi-Alkyl	5.47587
	A:TYR159 - A:PRO46	Pi-Alkyl	5.27588
	A:TRP22 - A:LYS318	Pi-Alkyl	5.08696
	A:TRP22 - A:LEU312	Pi-Alkyl	5.09244
	A:TRP22 - A:LYS318	Pi-Alkyl	5.08696
	A:TYR55 - A:LEU40	Pi-Alkyl	5.28065
	A:PHE70 - A:ALA21	Pi-Alkyl	5.09262
**6F86-Acidocin B**	A:ALA35:HN - A:GLY31:O	Conventional Hydrogen Bond	1.98751
	A:LEU38:HN - A:PHE34:O	Conventional Hydrogen Bond	1.91498
	A:GLY39:HN - A:ALA35:O	Conventional Hydrogen Bond	1.91582
	A:THR41:HN - A:LEU38:O	Conventional Hydrogen Bond	1.85722
	A:LEU42:HN - A:LEU38:O	Conventional Hydrogen Bond	2.1844
	A:LEU47:HN - A:ALA44:O	Conventional Hydrogen Bond	2.81403
	A:ALA50:HN - A:ALA46:O	Conventional Hydrogen Bond	1.87334
	A:ALA50:HN - A:LEU47:O	Conventional Hydrogen Bond	3.08033
	A:GLY51:HN - A:LEU47:O	Conventional Hydrogen Bond	2.04725
	A:LEU53:HN - A:ALA50:O	Conventional Hydrogen Bond	2.09438
	A:LEU38:CD1 - A:PHE34	Pi-Sigma	3.65395
	A:ALA35 - A:LEU47	Alkyl	4.5237
	A:LEU38 - A:LEU42	Alkyl	5.38259
	A:LEU38 - A:LEU47	Alkyl	5.23316
	A:ALA44 - A:VAL97	Alkyl	4.71877
	A:ALA46 - A:LEU38	Alkyl	4.37241
	A:ALA50 - A:ALA55	Alkyl	4.18352

**6F86-Lactacin F**	A:ARG19:HE - A:GLY69:O	Conventional Hydrogen Bond	2.10922
	A:ASN20:HN - A:GLY69:O	Conventional Hydrogen Bond	1.90757
	A:TRP60:HN - A:LEU56:O	Conventional Hydrogen Bond	1.96599
	A:TRP60:HN - A:PRO57:O	Conventional Hydrogen Bond	2.48877
	A:TRP60:HE1 - A:ASN25:O	Conventional Hydrogen Bond	2.0821
	A:THR64:HN - A:TRP60:O	Conventional Hydrogen Bond	2.47824
	A:THR64:HG1 - A:TRP60:O	Conventional Hydrogen Bond	1.82616
	A:GLY69:HN - A:THR64:O	Conventional Hydrogen Bond	2.31259
	A:GLY69:HN - A:THR67:OG1	Conventional Hydrogen Bond	2.76059
	A:PHE70:HN - A:THR64:O	Conventional Hydrogen Bond	1.95649
	A:ARG76:NH1 - A:PHE70	Pi-Cation	3.99269
	A:TRP60 - A:ALA29	Pi-Alkyl	4.99947
**2OH4-Acidocin B**	A:ARG1025:NH1 - A:ASP6:OD2	Attractive Charge	4.73116
	A:ARG1025:NH2 - A:ASP6:OD1	Attractive Charge	2.70258
	A:SER875:HG - A:GLY9:O	Conventional Hydrogen Bond	3.04026
	A:SER875:HG - A:ILE10:O	Conventional Hydrogen Bond	1.81591
	A:ARG878:HH11 - A:PHE8:O	Conventional Hydrogen Bond	1.7208
	A:ARG878:HH21 - A:PHE8:O	Conventional Hydrogen Bond	2.44669
	A:ARG878:HH21 - A:GLY9:O	Conventional Hydrogen Bond	1.79364
	A:SER882:HG - A:GLN7:O	Conventional Hydrogen Bond	2.83692
	A:ARG1025:HE - A:ASP6:OD2	Conventional Hydrogen Bond	1.96183
	A:ARG1025:HH11 - A:GLN7:OE1	Conventional Hydrogen Bond	1.90993
	A:ARG1025:HH21 - A:GLN7:OE1	Conventional Hydrogen Bond	2.08051
	A:LEU1067:HN - A:GLY54:O	Conventional Hydrogen Bond	2.018
	A:TYR1080:HH - A:THR56:O	Conventional Hydrogen Bond	2.26105
	A:TRP45:HE1 - A:ILE1051:O	Conventional Hydrogen Bond	2.54243
	A:ARG1078:CD - A:TYR2:OH	Carbon Hydrogen Bond	3.17524
	A:ASP1044:OD2 - A:TRP3	Pi-Anion	3.8465
	A:ASP1044:OD2 - A:TRP3	Pi-Anion	3.82295
	A:ILE10:CG2 - A:PHE843	Pi-Sigma	3.76951
	A:TYR1080 - A:TYR2	Pi-Pi Stacked	5.11477
	A:ALA879 - A:ILE10	Alkyl	3.84682
	A:ILE1051 - A:LEU42	Alkyl	4.49357
	A:LEU1065 - A:LEU53	Alkyl	4.85705
	A:ALA46 - A:ILE1051	Alkyl	4.84307
	A:ALA55 - A:PRO1066	Alkyl	4.53957
	A:ALA57 - A:PRO1066	Alkyl	4.87975
	A:ALA57 - A:MET1070	Alkyl	4.50432
	A:PHE8 - A:ALA879	Pi-Alkyl	5.24374

2OH4-Lactacin F	A:ARG817:HH12 - A:ALA65:O	Conventional Hydrogen Bond	2.60681
	A:ALA842:HN - A:TYR55:OH	Conventional Hydrogen Bond	2.52066
	A:ARG878:HH11 - A:ILE58:O	Conventional Hydrogen Bond	2.93861
	A:ARG878:HH11 - A:THR61:OG1	Conventional Hydrogen Bond	1.74232
	A:ARG878:HH21 - A:ILE58:O	Conventional Hydrogen Bond	1.7248
	A:LYS1021:HZ3 - A:GLN23:OE1	Conventional Hydrogen Bond	1.65013
	A:ARG1025:HH22 - A:VAL26:O	Conventional Hydrogen Bond	1.94758
	A:TRP22:HE1 - A:LYS1021:O	Conventional Hydrogen Bond	1.9774
	A:ASN25:HD21 - A:HIS814:NE2	Conventional Hydrogen Bond	2.19207
	A:TYR55:HH - A:LEU1047:O	Conventional Hydrogen Bond	1.89231
	A:LYS75:HZ3 - A:ARG817:O	Conventional Hydrogen Bond	1.68243
	A:HIS814:CE1 - A:PHE70:O	Carbon Hydrogen Bond	3.20234
	A:MET34:SD - A:THR1074:O	Sulfur-X	3.20017
	A:GLU816:OE2 - A:PHE70	Pi-Anion	4.38119
	A:CYS815:SG - A:PHE70	Pi-Donor Hydrogen Bond	3.77389
	A:CYS1022:SG - A:TRP22	Pi-Sulfur	5.85227
	A:ARG817 - A:ILE73	Alkyl	5.47757
	A:LEU818 - A:ILE73	Alkyl	5.04695
	A:PRO819 - A:LYS75	Alkyl	4.26888
	A:ALA879 - A:ILE58	Alkyl	4.21083
	A:ALA1048 - A:VAL50	Alkyl	5.06157
	A:PRO1066 - A:VAL50	Alkyl	4.93548
	A:ARG1078 - A:MET34	Alkyl	4.39874
	A:PRO46 - A:LEU1065	Alkyl	4.69462
	A:ALA49 - A:PRO1066	Alkyl	4.65588
	A:VAL50 - A:ILE1051	Alkyl	4.76826
	A:ALA51 - A:ILE1051	Alkyl	3.66258
	A:PHE843 - A:ILE58	Pi-Alkyl	5.30278
	A:TRP22 - A:LYS1021	Pi-Alkyl	4.53356
	A:HIS54 - A:ALA1048	Pi-Alkyl	4.58902
	A:TYR55 - A:ALA842	Pi-Alkyl	4.88129
	A:TYR55 - A:ALA1048	Pi-Alkyl	4.38572
